# Synthesis, Characterization and Biological Activities of Cu(II), Co(II), Mn(II), Fe(II), and UO_2_(VI) Complexes with a New Schiff Base Hydrazone: *O*-Hydroxyacetophenone-7-chloro-4-quinoline Hydrazone

**DOI:** 10.3390/molecules16108629

**Published:** 2011-10-13

**Authors:** Nora H. Al-Shaalan

**Affiliations:** Chemistry Department, College of Science, Princess Nora Bint Abdul Rahman University, 11322 Riyadh, P.O. 240549, Saudi Arabia; Email: nora_shaalan@yahoo.com; Fax: +096-612489254

**Keywords:** Schiff base ligand, metal complexes, antimicrobial, IR spectra, UV-Vis spectra

## Abstract

The Schiff base hydrazone ligand **HL** was prepared by the condensation reaction of 7-chloro-4-quinoline with *o*-hydroxyacetophenone. The ligand behaves either as monobasic bidentate or dibasic tridentate and contain ONN coordination sites. This was accounted for be the presence in the ligand of a phenolic azomethine and imine groups. It reacts with Cu(II), Ni(II), Co(II), Mn(II), UO_2_ (VI) and Fe(II) to form either mono- or binuclear complexes. The ligand and its metal complexes were characterized by elemental analyses, IR, NMR, Mass, and UV-Visible spectra. The magnetic moments and electrical conductance of the complexes were also determined. The Co(II), Ni(II) and UO_2_ (VI) complexes are mononuclear and coordinated to NO sites of two ligand molecules. The Cu(II) complex has a square-planar geometry distorted towards tetrahedral, the Ni(II) complex is octahedral while the UO_2_ (VI) complex has its favoured heptacoordination. The Co(II), Mn(II) complexes and also other Ni(II) and Fe(III) complexes, which were obtained in the presence of Li(OH) as deprotonating agent, are binuclear and coordinated via the NNNO sites of two ligand molecules. All the binuclear complexes have octahedral geometries and their magnetic moments are quite low compared to the calculated value for two metal ions complexes and thus antiferromagnetic interactions between the two adjacent metal ions. The ligand **HL** and metal complexes were tested against a strain of Gram +ve bacteria (*Staphylococcus aureus*), Gram −ve bacteria (*Escherichia coli*), and fungi (*Candida albicans*). The tested compounds exhibited high antibacterial activities.

## 1. Introduction

Schiff bases play an important role in inorganic chemistry as they easily form stable complexes with most transition metal ions. The development of the field of bioinorganic chemistry has increased the interest in Schiff base complexes, since it has been recognized that many of these complexes may serve as models for biologically important species [1,2,3,4,5]. The remarkable biological activity of the acid hydrazide (R–CO–NH–NH_2_) class of Schiff base, their corresponding aroylhydrazones (R–CO–NH–N=CH–R) and the dependence of their mode of chelation with transition metal ions present in the living systems have been of significant interest [6,7,8,9,10,11]. The coordination compounds of aroyl-hydrazones have been reported to act as enzyme inhibitors and are useful due to their pharmacological applications [6,7,8,9,10,11]. Isonicotinic acid hydrazide (INH) is a drug of proven therapeutic importance and is used for bacterial ailments, e.g., tuberculosis. Hydrazones derived from condensation of isonicotinic acid hydrazide with pyridine aldehydes have been found to show better antitubercular activity than INH [12]. Agarwa investigated the coordinating ability of INH-derivatives with metal ions [13]. Tri- and terdentate Schiff bases may contain ONO or ONS donor atoms. Their metal complexes may be monomeric, dimeric, trimeric or tetrameric with abnormal magnetic properties and characteristic structures [14,15,16]. In the present investigation we describe the synthesis, characterization, and antimicrobial activity of transition metal complexes of a novel Schiff’s base hydrazone containing the quinoline moiety; *o*-hydroxyacetophenone-7-chloro-4-quinoline hydrazine (**HL**).

## 2. Results and Discussion

### 2.1. Characterization of the Ligand

**Figure 1:**
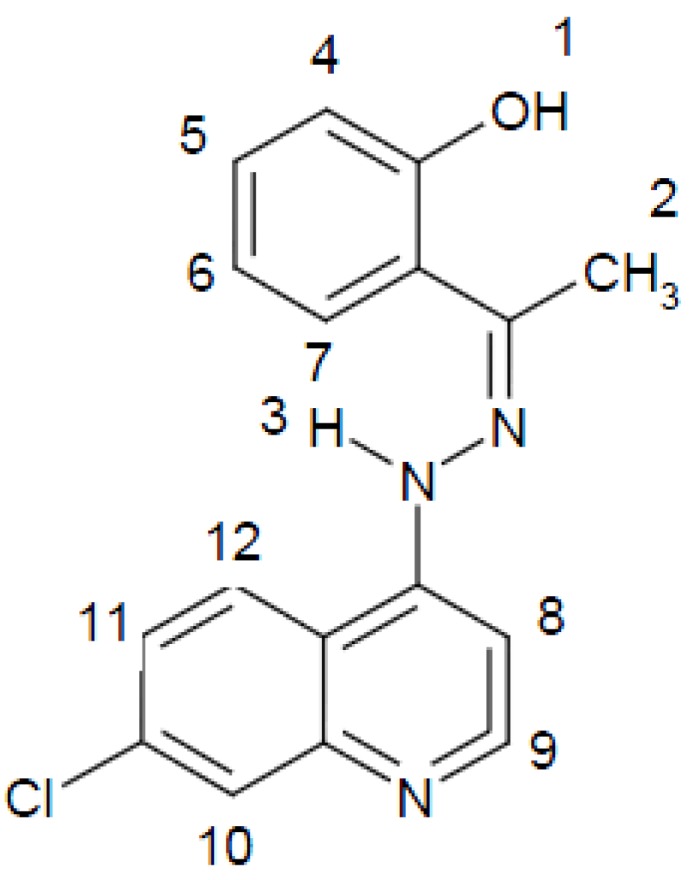
Structure and atom numbering of *o*-hydroxyacetophenone-7-chloro-4-quinolinehydrazine (**HL**).

The organic ligand **HL** (Figure 1) was prepared by reacting *o*-hydroxyacetophenone with 7-chloro-4-hydrazinoquinoline in the molar ratio 1:1.

**Table 1:** Elemental analyses, color, yield, melting points and molar conductance of HL and its corresponding metal complexes.

Compound	F.W.	Color	Yield(%)	M.P. (°C)	Elemental analysis, Found / (Calcd) %					Solubility
					C	H	N	CL	M	
C_17_H_14_N_3_OCl (**HL**)	311.5	Deep Orange	66	225	65.37 (65.49)	4.39 (4.49)	13.48 (13.48)	11.30 (11.4)	--	Soluble in most common organic solvent
[(HL)_2_Cu].EtOH (**1**) C_36_H_32_N_6_O_3_Cl_2_Cu	731	Green	33	287	59.00 (59.10)	4.27 (4.38)	11.29 (11.49)	9.51 (9.71)	8.66 (8.76)	Soluble in acetone and insoluble in methanol and ethanol
[Ni (HL)_2_(OH_2_)_2_] 2EtOH (**2**) C_28_H_42_N_6_O_6_Cl_2_Ni	808	Pale Green	25	220	56.24 (56.44)	5.13 (5.20)	10.40 (10.40)	8.79 (8.79)	7.30 (7.30)	Soluble in most common organic solvent
[(L)_2_Ni_2_(OH_2_)_6_]^a ^(**3**) C_34_H_36_N_6_O_8_Cl_2_Ni_2_	845	Yellowish Green	47	250 (decomp)	48.15 (48.28)	4.07 (4.26)	9.75 (9.94)	8.08 (8.28)	13.67 (13.96)	Soluble in acetone and insoluble in methanol and ethanol
[(L)_2_Co_2_(OH_2_)_6_]^a ^(**4**) C_34_H_36_N_6_O_8_Cl_2_Co_2_	845	Brown	34	260 (decomp)	48.00 (48.18)	4.50 (4.28)	10.12 (9.99)	8.07 (8.26)	13.79 (13.98)	Soluble in most common organic solvent except diethyl ether
[(L)_2_Mn(OH_2_)_6_]^a ^(**5**) C_34_H_36_N_6_O_8_Cl_2_Mn_2_	837	Deep brown	48	225	49.00 (48.75)	4.50 (4.30)	9.10 (10.04)	8.27 (8.36)	12.97 (13.14)	Soluble in acetone and insoluble in methanol and ethanol and ether
[(HL)_2_UO_2_(EtOH)] (**6**) C_36_H_32_N_6_O_5_Cl_2_U	937	Red	75	225 (decomp)	46.31 (46.10)	3.51 (3.42)	9.21 (8.96)	7.73 (7.58)	25.20 (25.40)	Soluble in most common organic solvent except diethylether
[(L)_2_Fe_2_Cl_2_(OH_2_)_4_] 2EtOH^a ^(**7**) C_38_H_44_N_6_O_8_Cl_4_Fe_2_	966	Reddish brown	55	250 (decomp)	47.35 (47.20)	4.26 (4.55)	8.58 (8.70)	14.52 (14.70)	11.45 (11.59)	Soluble in most common organic solvent except diethylether

^a^ Obtained from the reaction of the ligand in presence of LiOH; ^b^ measured at ambient temperature for 1 × 10^−3^ M in DMF.

Elemental analyses (Table 1) of the ligand reflected that the ligand has the molecular formula given. The ^1^H-NMR spectrum (Table 2) of the ligand in deuterated dimethylformamide showed two D_2_O exchangeable signals at δ 14.5 and 11.7 ppm for the protons of the phenolic OH and the NH groups, respectively [16,17]. A signal is also observed at δ 2.4 ppm for the –CH_3_ group [17,18].

**Table 2:** ^1^H-NMR data of the ligand **HL** in DMF-d_6_.

Chemical shift, δ_TMS_ (ppm)	Assignment ^a^
14.5	[s, 1H] (1)
2.4	[s, 3H] (2)
11.7	[s, 1H] (3)
7.9–8.3	[m, 9H, Ar–H and quinoline–H]

**Table 3:** Characteristic IR bands (cm^−1^) of the HL and its corresponding metal complexes.

Compound	ν(C=N)	ν(N-H)	ν(N-N)	ν(M-N)	ν(M-O)	ν(OH), H_2_O or alcohol	Other bands
**HL**	1524 s	3300 m	1140s	---	---	3530m, br (νOH-phenolic)	1278 (δ OH-phenolic)
[(HL)_2_Cu] EtOH (1)	1520 s	3215 m	1136 s	420 w	540 m	3426 m, br (νOH-alcohol	---
[(HL)_2_Ni(OH_ 2_)_2_] 2EtOH (**2**)	1510 m	3200 s	1137 w	425 w	520 m	3440 m, br (νOH-coordinated water over-lapped with (νOH- alcohol	---
[(L)_2_Ni_2_(OH_2_)_6_] (**3**)	1514 sh	---	1125 w	410 w	515 m	3436 m, br (νOH-coordinated water	---
[(L)_2_Co_2_(OH)_2_)_6_] (**4**)	1527 sh	---	1136 S	445W	520W	3438 m, br (νOH-coordinated water	---
[(L)_2_Mn(OH_2_)_6_] (**5**)	1507 m	---	1137 s	465 w	520 m	3439 m, br (νOH-coordinated water	---
[(HL)_2_UO_2_(EtOH)] (**6**)	1505	3214 sh	1134 s	460 w	540 w	3435 m, br (νOH-coordinated water over-lapped with (νOH-coordinated alcohol	901 s ν_3_(O=U=O
[(L)_2_Fe_2_Cl_2_(OH_2_)_4_] 2EtOH (**7**)	1515 s	---	1138 W	460 W	515 W	3435 m, br (νOH-coordinated water overlapped with (νOH- alcohol	---

s: strong, w: weak, m: medium, br: broad, sh: shoulder.

**Figure 2:**
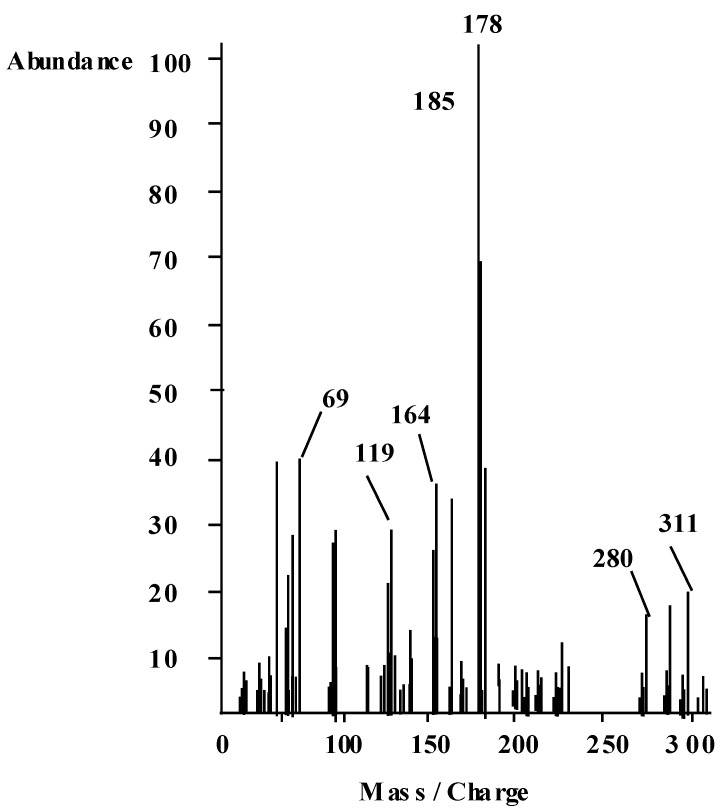
Mass spectrum of HL ligand recorded at 300 °C and 70 eV.

**Figure 3:**
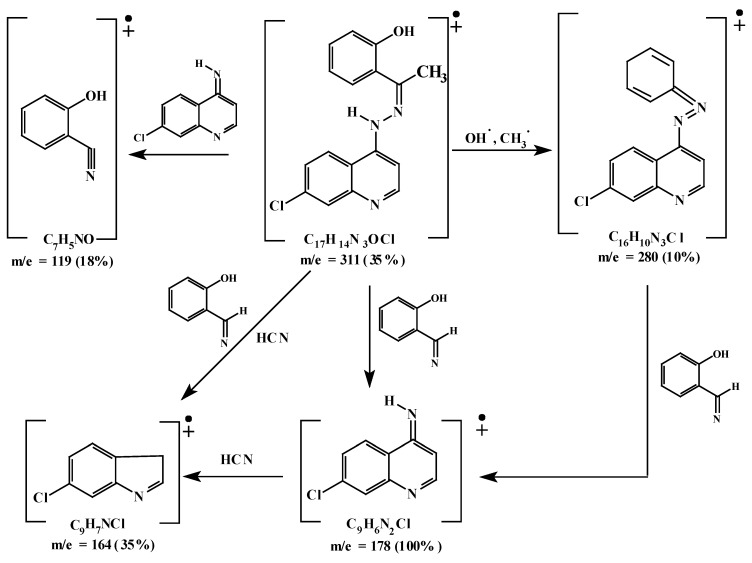
Mass fragmentation pattern of the ligand **HL**.

The UV-VIS spectrum (Table 4) recorded for the ethanolic solution of the ligand showed absorption bands at 48,067 and 44,642 cm^−1^ assigned for π-π * transitions within the aromatic and quinoline rings. The band observed at 36,231 cm^−1^ would be due to the n-π *transition of the C=N group. The absorption bands at 26,176 and 24,752 cm^−1^ are assigned to CT transitions. The band at 24,752 cm^−1^ encroaches on the visible region and impacts the ligand its color [19,20]. The mass spectrum of the ligand (Figure 2) showed its molecular ion at *m/z* 311, which coincide with its formula weight. Metastable ion(s) are not observed [18]. Figure 3 represents the proposed fragmentation pattern of the ligand.

**Table 4:** Magnetic moment, electronic and conductance measurements spectral data (cm^−1^) for HL and its metal complexes ^a^.

Compound	_μeff__._^b^ B.M.	_μcomble_^c^ found (expected ^d^)B.M.	*π→π, n→π* and charge transfer transitions	d→dTransition(cm^−1^)	EC ^f^
HL	---	---	48067, 44642, 36231, 26176,24752	---	---
[(HL)_2_Cu] EtOH (**1**)	1.807	1.807	36765 ,28612, 22396	15850	2.4
[HL)_2_Ni(OH_ 2_)_2_] 2EtOH (**2**)	2.85	2.85	37037, 28653, 22371	15850	2.0
[(L)_2_Ni_2_(OH_2_)_6_] (3)	---	4.44 (5.71)	37437, 28670,22300	1518.45, 11957	2.6
[(L)_2_Co_2_(OH)_2_)_6_] (4)	---	7.12 1(10.4)	370307, 28600, 22271	15250, 15390	2.4
[(L)_2_Mn(OH_2_)_6_] (5)	---	5.39 (8.40)	37085, 28500, 22471	21331.9, 11733.33	3.6
[(HL)_2_UO_2_(EtOH)] (6)	---	---	37537, 27853, 22871	22500, 19040	1.5
[(L)_2_Fe_2_Cl_2_(OH_2_)_4_] 2EtOH (7)	---	7.49 (10.80)	37137, 27653, 22571	14331, 13850	68

^a^ measured as Nujol mull. _bµeff__._ is the magnetic moment of only one cationic species in the complex. ^c^ µ_effl_ is the magbetic moment of all cations in the complex only one cationic species in the complex. ^d^ The expected values were calculated by adding known susceptibilities of the metal cations present in the complex in the suggested structures. ^f^ EC=Electrical conductance, 10^−3^ M solution in DMF, Ohm^−1^ cm^2^ mol^−1^.

Elemental analyses and IR spectra of the ligand and its metal complexes (Table 1 and Table 3) showed that the HL coordinated with the metal ions either as monobasic bidentate or dibasic ions either as monobasic bidentate or dibasic.

### 2.2. Metal Complexes

The Schiff base hydrazone ligand **HL** containing ON or ONN coordination sites behaves as either monobasic bidentate or dibasic tridentate, respectively. The ligand was reacted with Cu(II), Ni(II), Co(II), Mn(II), UO_2_(VI) and Fe(III) ions to yield either mononuclear or binuclear metal complexes as listed in Table 1 together with their chemical analysis and other physical data.

Mono- and binuclear Ni (II) complexes were obtained without and with the addition of LiOH as a deprotonating agent, respectively. Table 3 shows the characteristic IR bands of the ligand and its metal complexes. Table 4 shows the magnetic moments, conductance, UV-VIS bands of the complexes. The chemical analyses, UV-VIS and IR bands of the parent ligand are also included for comparison purposes.

### 2.3. IR Spectra of the Metal Complexes

The IR spectra of the mononuclear Cu(II) and Ni(II), complexes (1) and (2), respectively (Table 3) showed that the band due to the phenolic OH group that appeared in the spectrum of the ligand at 3,530 cm^−1^ had disappeared in the spectra of these complexes. This may be due to the displacement of its proton by the metal ion. Moreover, the spectra showed that the vibrations of the –C=N group were shifted to a lower frequency due to the coordination of the nitrogen atom of the azomethine group. As a result, the Schiff base hydazone ligand **HL** coordinates in these complexes as a monobasic bidentate, ON, ligand via the oxygen atom of the phenolic OH and the nitrogen atom of the azomethine groups [21].

The IR spectra of the binuclear Ni (II), Co(II) and Mn(II) complexes **3–5** (Table 3) showed a broad band at ≈3,440–4,000 cm^−1^ due to ν(OH) of the coordinated water molecules which replaces the band of the phenolic OH groups observed in the spectrum of the parent ligand. Moreover, the bands due to the imine NH group are absent in the spectra of the complexes. The bands due the –C=N group are shifted to lower frequencies. These results indicate the replacement of the hydrogen ions of both the phenolic and imine groups by the metal cations. As a result, the Schiff base hydrazone ligand **HL** coordinates in these complexes as a diprotic tridentate ONN ligand via the oxygen atom of the phenolic OH, the nitrogen atom of the azomethine and the nitrogen atom of the imine groups.

The IR spectrum of the mononuclear UO_2_(VI) complex **6** (Table 3) showed a broad band at 3,435 cm^−1^ assigned to ν(OH) of the coordinated ethanol group. ν(NH) of the uncoordinated NH groups appeared as a shoulder at 3,214 cm^−1^, exactly at the same frequency as for the parent ligand. However, no splitting of this band was observed, which may be due to the larger separation of the two ligand molecules, due to the larger volume of the UO_2_(VI) cation in the complex molecules. A well-characterized band appeared at 1,505 cm^−1^ assigned to the coordinated –C=N group. The band occurs at a lower frequency compared to that of the parent ligand indicating its involvement in coordinating the UO_2_(VI) cation in addition to the phenolic oxygen atoms after replacing its hydrogen ions by the uranyl (VI) cation. The ν_3_ (O=U=O) appeared as a strong band at 901 cm^−1^ overlapping with another band already present in the spectrum of the parent ligand and thus gaining higher intensity [21,22].

The IR spectrum of the binuclear Fe (III) complex **7** (Table 3), showed a broad band at 3,435 cm^−1^ due to ν(OH)of the outer sphere ethanol molecules. Another band appeared at 1,515 cm^−1^. This band is shifted to lower frequency compared to that of the parent ligand. This shift would be due to the effect of the tripositive ferric ion on decreasing the force constant of the –C=N bonds. However, the splitting observed would be due to lattice effects which would lead to non-equivalent position of these bans indicating a bridged binuclear complex [21,22]. New bands appeared in the spectra of all the complexes at 515–540 and 410–465 cm^−1^ that would be assigned to νM-O and νM-N, respectively.

### 2.4. Magnetic Moments and Electronic Spectral Data of the Metal Complexes

The electronic spectra and magnetic moments of the metal complexes are listed in Table 4. Generally, in all spectra of metal complexes, the absorption bands due to π-π* and n-π *transitions that observed in the spectrum of the free ligand higher than 22,300 cm^−1^ have shifted to lower frequencies due to the coordination of the ligand with metal ions.

The spectra of the mononuclear cu(II) complex (1) (Table 4) showed absorption bands at 15,520–15,850 cm^−1^ which could be attributed to the ^2^A_1g_→^2^B_1g_ transitions characterized Cu(II) ion in a square-planar geometry [23]. The square-planar geometry of Cu(II) ion in the complex is confirmed by the measured magnetic moments values, 1.807 B.M. The square-planar geometry is achieved by the coordination of two molecules of HL each as monobasic bidentate ligand, to the copper(II) ion [23,24,25]. The shift of the absorption band to lower energy than that expected for square-planar geometry, at 18,191.8 cm^−1^ for square-planar *N,N'-*ethylenebis(salicylideneimine) copper(II), Cu (acacen) [25] may be due to the distortion of the square-planar geometry towards tetrahedral [23,24,25].

The electronic spectrum of the mononuclear Ni(II) complex (2), Table 4, showed broad absorption band at 15,850 cm^−1^ which may be assigned to ^3^A_1g_(F)→^3^T_2g_(F), while the absorption due to ^3^A_2g_(F)→^3^T_1g_(p) is overlapped with the ligand absorption bands. This indicates that the Ni (II) ion coordinated to N_2_O_2_ sites in an octahedral geometry [20,22]. The Ni(II) ion complete its six-coordination sphere by two water molecules. The third transition due to ^3^A_2g_(F)→^3^T_1g_ (p) would be out of the scale of the used spectrophotometer. The magnetic moment of the complex is 2.85 B.M. which agrees with the presence of Ni(II) ion in octahedral geometry [21,23]. The spectrum of the binuclear Ni(II) complex (3) showed a band in the range 15,184.5–11,957.5 cm^−1^. This band may be due to the ^3^A_2g_→^3^T_1g_ electronic transitions of both Ni (II) cations in an octahedral geometry. The magnetic moment of this complex is 4.44, which is smaller than the calculated value for two Ni(II) ions in octahedral geometries and may indicate antiferromagnetic interactions between adjacent Ni(II) ions in the complex.

Octahedral, tetrahedral and square-planar cobalt (II) complexes show magnetic moment between 4.7–5.2, 4.2–4.8 and 2.2–2.9 B.M., respectively [23]. The μ_eff_ value measured for the present Co(II) complex (4), Table 4, is 7.12 B.M. The magnetic moment of the complex is 7.52 B.M. which indicates that both Co(II) ions are present in octahedral geometries. The electronic spectrum of the complex showed two d-d transitions in the range 15,250–15,390 cm^−1^ and 18,410–18,480 cm^−1^ due to the ^4^T_1g_(F) → ^4^A_2g_(F) and ^4^T_1g_ (F)→^4^A_2g_(p) transitions, respectively [13]. This indicates an octahedral configuration around Co(II) ions. The ^4^T_2g_(F)→^4^T_1g_(F) transition (9.510 cm^−1^) would be observed in near IR region. This region is outside the range of our spectrophotometer [23].

The spectrum of the binuclear Mn (II) complex (5) showed a series of weak bands in the range 21,331.96 11,733.33 cm^−1^. These bands are both Laporte and spin-forbidden. However, due to instantaneous distortion of the octahedral structures around the metal cations, weak bands sometimes appear [21,23]. The magnetic moment of the complex is 5.39 B.M. and indicates antiferromagnetic interaction between the adjacent metal cations.

The electronic spectra of the diamagnetic uranyl complex(6) show two bands, in addition to the ligand bands The first band observed at 22,500 cm^−1^ corresponding to charge transfer from equatorial donor atoms of the ligand to the uranyl ion. The second band observed at 19,040 cm^−1^ due to electronic transitions from apical oxygen atom to the f- orbitals of the uranyl atom characteristic of the uranyl moiety.

On the other hand, the electronic spectra of Fe(II) complex(7) showed broad bands 14,331 and at 13,850 cm^−1^. The former band may be due to the spin forbidden transition ^6^A_1g_ →^4^T_2g_(G), which may gain intensity as a result of the vibronic mechanism in the octahedral field around ferric ion. The second bands may be attributed to ^6^A_1_→^4^T_1_(G) transitions [23]. In addition, a third absorption band with high intensity observed at 25,252 cm^−1^ assigned to a charge transfer transition. The magnetic moment of the complex is 7.49 B.M. This value is quite low compared to the calculated magnetic moment value for binuclear ion complexes [23].

**Figure 4:**
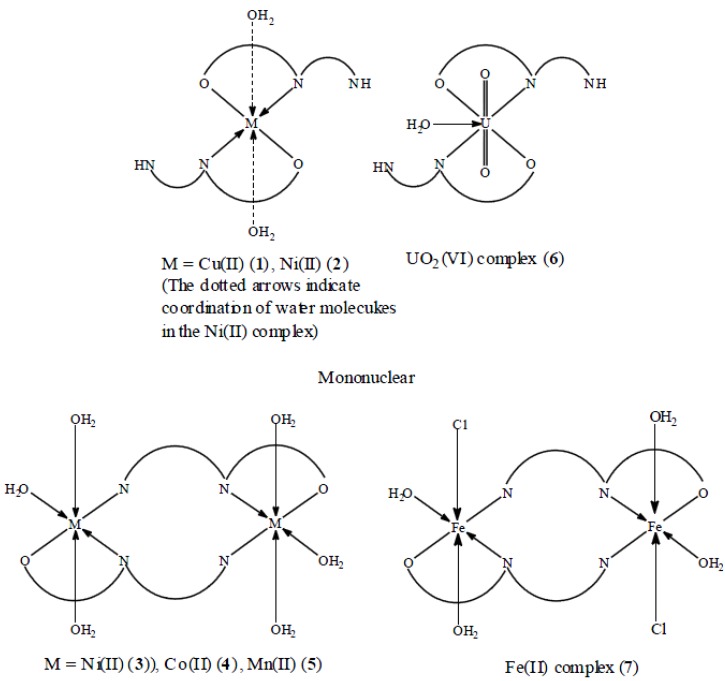
Suggested structures of the metal complexes of the ligand.

### 2.5. Molar Conductance of the Metal Complexes

The conductance measurements, recorded for 10^−3^ M solutions of the metal complexes in DMF, are listed in Table 1. All complexes are non-conducting, indicating their neutrality and that the divalent cations have replaced the phenolic and/ or the imine protons, However, the Fe(III) complex **7** showed an appreciable amount of conductance, may be through the replacement of a part of the coordinated chloride ions by solvent molecules as previously reported [26], a phenomenon usually encountered in complexes containing chloride ions. Based on the above results the structures in Figure 4 are suggested for the metal complexes.

### 2.6. ^1^H-NMR Spectrum of the Uranyl Complex

The uranyl complex **6** was selected as it is diamagnetic. Its ^1^H-NMR spectrum in DMF and after deuteration are discussed. The spectrum of the complex differs from that of the free ligand in the following aspects:

(1). The disappearance of the signal due to the phenolic OH group is attributed to its involvement in coordinating the uranylcation, While the signal due to the NH group was broad and appeared at δ = 12.4 ppm compared to that of the ligand which appeared at, δ =11.7 ppm, *i.e.*, shifted to low-field.
(2). The signals due to the aromatic ring showed fine structure and appear as four separate signals at δ = 7.14, 7.53 and 7.6 ppm.
(3). The NH group which did not take part in coordinating the uranylcation disappeared on deuteration.
(4). The CH_3_ group signal remained unchanged as observed in the parent ligand.

### 2.7. Antimicrobial Activity and Minimum Inhibitory Concentration (MIC)

The test was performed according to the reported previously method [27]. The tests were carried for concentrations of 1, 25, 50, 100% in DMSO of the compounds. The inhibition zones caused by the various compounds on the microorganisms were examined. The results of the preliminary screening test are listed in Table 5. From the data, it is clear that 7-chloro-4-hydrazinoquinoline was highly active against all organisms and at all concentrations while the *o*-hydroxyacetophenone-7-chloro-4-quinoline hydrazone ligand (**HL**) exhibited activity at its 25, 50 and 100% conc. for *Staphylococcus aureus* and at 100% for *Escherichia coli* and also exhibited activity at its 25, 50 and 100% conc. for *Candida albicans*. Remarkable result is that the complexes, [(L)2Ni2(OH2)6](**3**), [(L)2CO2(OH2)6](**4**), [(L)2Mn(OH2)6](**5**) were found to have high activity against all stains in all of their concentrations except their 1% concentration. On the other hand, [(HL)_2_UO_2_(EtOH)](**6**) was found to have higher activity against the strains *Staphylococcus aureus* and *Candida albicans* at its 25, 50, and 100% concentrations, while for *Escherichia coli*, activity was only exhibited for 100% concentration. Mononuclear complexes [(HL)_2_Cu] EtOH (**1**) and [(HL)(**2**) only exhibited activity at 100% conc. for all tested strains. The tested compounds showed an inhibitory activity of 25%.

**Table 5:** Results of anti-microbial activity of some tested complexes.

Compound	Conc.%	Micro-organism		
		*S. aureus* ATCC * 6538MIC *** 25%	*E. coli* ATCC 8739 MIC 25%	*C. albicans* ATCC 10231MIC 25%
*o*-Hydroxyacetophenone-7-chloro-4-quinoline hydrazine (**HL**)	1 **			
	**25**	+ve	−ve	+ve
	50	+ve	−ve	+ve
	100	+ve	+ve	+ve
[(HL)_2_Cu] EtOH (**1**)	1	+ve	+ve	+ve
	25	+ve	+ve	+ve
	50	+ve	+ve	−ve
	100	−ve	−ve	−ve
[HL)_2_Ni(OH_2_)_2_] 2EtOH (**2**)	1	+ve	−ve	−ve
	25	+ve	+ve	+ve
	50	+ve	+ve	+ve
	100	−ve	−ve	−ve
[(L)_2_Ni_2_(OH_2_)_6_] (**3**)	1 **			
	25	−ve	+ve	−ve
	50	−ve	−ve	−ve
	100	−ve	−ve	−ve
[(L)_2_Co_2_(OH_2_)_6_] (**4**)	1	+ve	+ve	+ve
	25	−ve	+ve	−ve
	50	−ve	−ve	−ve
	100	−ve	−ve	−ve
[(L)_2_Mn(OH_2_)_6_] (**5**)	1 **			
	25	−ve	+ve	−ve
	50	−ve	−ve	−ve
	100	−ve	−ve	−ve
[(HL)_2_UO_2_(EtOH)] (**6**)	1	+ve	+ve	+ve
	25	−ve	+ve	−ve
	50	−ve	+ve	−ve
	100	−ve	−ve	−ve
[(L)_2_Fe_2_Cl_2_(OH_2_)_4_].2EtOH (**7**)	1	+ve	+ve	+ve
	25	−ve	+ve	−ve
	50	−ve	−ve	−ve
	100	−ve	−ve	−ve
7-Chloro-4-hydrazinoquinoline	1	+ve	+ve	+ve
	25	−ve	−ve	−ve
	50	−ve	−ve	−ve
	100	−ve	−ve	−ve

* number of strain in the American collection; ** not tested in this concentration; *** MIC is the lowest concentration of the product material solution to inhibit the growth of the microorganism key to symbols: Active and inhibit the growth of the stain = −ve; Inactive = +ve.

## 3. Experimental

### 3.1. General

Copper(II) acetate monohydrate, nickel(II) acetate tetrahydrate, uranyl (VI) acetate dihydrate, iron(III) chloride hexahydrate, cobalt(II) acetate tetrahydrate, manganese(II)acetate tetrahydrate, zinc (II) dihydrate and lithium hydroxide monohydrate were obtained from BDH. *o*-Hydroxyacetophenone, salicylaldehyde and hydrazine hydrate (100%), were either BDH or Merck products. Organic solvents used were reagent grade. 4,7-Dichloroquinoline was obtained as a commercial sample from EI-Nasr-Company for Pharmaceutical Chemicals (Cairo, Egypt), and was purified in the laboratory as follows: Under warming and constant stirring, 10 g (10 mmol) of the sample were dissolved in absolute ethanol (150 mL). The solution was filtered while hot. After cooling, white cottony crystals were formed which were filtered off and washed with ethanol (5 mL). The product was crystallized from ethanol. The formed crystals were stored in a brown container to avoid light, which may affect it.

### 3.2. Synthesis of the Schiff Base Hydrazone Ligand *(**HL**)*

#### 3.2.1. Synthesis of 7-Chloro-4-hydrazinoquinoline

100% Hydrazine hydrate (25 mL, 50 mmol, dissolved in 30 mL absolute ethanol) was added gradually to 4,7-dichloroquinoline (10 g, 5 mmol, dissolved in 20 mL ethanol). The mixture was refluxed for 2 h. After 1/2 h, a golden yellow precipitate started to form. After the refluxing time was reached, the mixture was allowed to cool for 6 h. The golden yellow precipitate was filtered and washed with absolute ethanol (5 mL) and re-crystallized from absolute ethanol.

#### 3.2.2. Synthesis of the ligand **HL**

The ligand **HL** was prepared by adding *o*-hydroxyacetophenone (1.5 g, 1.1 mmol, dissolved in 10 mL absolute ethanol) to 7-chloro-4-hydrazinoquinoline, (1.8 g, 1 mmol dissolved in 10 mL absolute ethanol). The reaction mixture was stirred thoroughly and refluxed for 2 h, and the precipitated golden orange crystals were filtered and washed with a few drops of ethanol.

### 3.3. Synthesis of the Metal Complexes

The reaction of a solution of the metal salt was tried first with the ligand solution. If a product was obtained the same reaction was tried again after prior deprotonation of the ligand with LiOH. In most cases, the same yield was obtained except in the case of the Ni(II) salt. However, in other reactions deprotonation was a must in order to obtain a product.

#### 3.3.1. Cu(II) Complex, [(HL)_2_Cu] EtOH

Cu(II) acetate salt (0.19 g, 0.95 mmol) was dissolved in ethanol (10 mL) and heated on a water bath to ensure complete dissolution of the salt. The metal salt solution was added gradually to a stirred solution of the ligand (0.06 g, 1.9 mol dissolved in 10 mL ethanol). The mixture was further stirred for 10 h to ensure complete precipitation of the green complex. The precipitated solid was filtered, washed with ethanol (5 mL), then dried in air and collected.

#### 3.3.2. Ni(II) Complexes

The reaction of nickel(II) acetate with the ligand yielded two different products in the presence or absence of LiOH.

##### 3.3.2.1. In the Absence of LiOH, [(HL)_2_Ni(OH_2_)_2_] 2EtOH

Ni(II) acetate salt (0.34 g, 1.2 mmol) was dissolved in ethanol (10 mL) and added to the ligand solution (0.8 g, 2.4 mmol, dissolved in 10 mL ethanol). The color changes from yellow to green during the addition of the salt solution and stirring was continued for 6 h. A pale green precipitate was obtained which was filtered off, washed with ethanol (5 mL), then dried in air.

##### 3.3.2.2. In the Presence of LiOH, [(L)_2_Ni(OH_2_)_6_]

LiOH (0.1 g, 2.4 mmol) was dissolved in methanol (5 mL) and added dropwise to a stirred solution of the ligand (0.8 g > 2.4 mmol dissolved in 5 mL methanol). A nickel(II) acetate solution (0.34 g, 1.2 mmol dissolved in 10 mL methanol) was added gradually to the stirred solution of the lithium salt of the ligand. The color of the solution changed from yellow to green. Stirring was continued for 6 h when a yellowish green precipitate was formed which was filtered off and washed with small amounts of methanol and then dried in air.

#### 3.3.3. Co(II) Complex, [[(L)_2_CO_2_(OH_2_)_6_]

Co(II) acetate (0.3 g, 1.2 mmol) was dissolved in ethanol (10 mL). The metal salt solution was added gradually to a stirred solution of the ligand (0.8 g, 2.4 mmol dissolved in 10 mL ethanol). The reaction solution was further stirred for 10 h when a brown precipitate was formed which was filtered off and washed with small amounts of ethanol and then dried in air.

#### 3.3.4. Mn(II)Complex, [(L)_2_Mn_2_(OH_2_)_6_]

LiOH (0.1 g, 2.4 mmol) was dissolved in methanol (5 mL) and added to the ligand solution (0.8 g, 2.4 mmol, dissolved in 5 mL methanol). A solution of the metal salt (0.3 g, 1.2 mmol, in 10 mL methanol) was added gradually to the stirred lithium salt solution of the ligand. During the addition, the color of the solution changed from yellow to brown precipitate began to form. Stirring was continued for further 4 h and the solution was let stand overnight at room temperature. The brown solid was filtered off, washed with small amounts of methanol, then diethyl ether was added and the product was dried in air.

#### 3.3.5. UO_2_(VI) Complex, [(HL)_2_UO_2_(EtOH)]

UO_2_(VI) acetate (0.5 g, 1.2 mmol) was dissolved in 10 mL H2O and added gradually to the ligand solution (0.8 g, 2.4 mmol, dissolved in 10 mL ethanol). A red precipitate was formed and stirring was continued further for 4 h. The product was filtered off, washed with small amounts of H_2_O and then dried in air.

#### 3.3.6. Fe(III) Complex, [(L)_2_Fe_2_C_l2_(OH)_4_] EtOH

LiOH (0.1 g, 2.4 mmol) was dissolved in methanol (5 mL) and added to the ligand solution (0.8 g, 2.4 mmol). A solution of the metal salt (0.33 g, 1.2 mmol, in 10 mL methanol) was added gradually to the stirred lithium salt solution of the ligand. During the addition, the color of the solution changed from brown and a brown precipitate began to form. Stirring was continued for further 4 h and the solution was let stand overnight at room temperature. The brown solid was filtered off, washed with small amounts of methanol, then diethyl ether was added and the product was dried in air.

### 3.4. Synthesis of Samples for Microbiological Analysis

0.02 g of each complex was dissolved in 100 mL dimethylsulfoxide, DMSO, to produce 0.02% solutions. To aeries of culture tubes in which each tube containing sterile 5 mL double strength solution of Soyabean Casein Digest Medium (Tryptic soy Broth), 5 mL of the 0.02% DMS solution was added to each tube and mixed. To determine the bacteriostatic efficiency of the Staphylococcus aureu and Escherichia coli organism, 1 mL of a 1:10 dilutioned solution of Tryptic soy Broth (TSB) was added to each culture tube. A 1:10 diluted solution of Tryptic Soy Broth (TSB) was prepared by pipetting 1 mL of bacterial cultures incubated at 37 °C for 24 h into 9 mL of sterile Tryptic Soy Broth. For the test of the fungistatical efficiency, 0.1 mL of undiluted sample incubated for 72 h at 37 °C TSB cultivation efficiency, 0.1 mL of undiluted inoculated culture tubes were inoculated at 30–35 °C for 18–24 h. After that, the “MIC” level was assessed visually. MIC was recorded as the first clear tubes after turbidity, starting with the blank broth. In other words, the highest dilution of the antiseptic/disinfectant preventing growth is taken as the “MIC” of the test organism [5,26].

### 3.5. Physical Measurements and Analysis

Electronic spectra recorded for solution of the ligand, **HL** in DMF, and for the metal complexes as Nujol mull on a Jasco UV-VIS spectrophotometer model V-550 UV-VIS. The IR spectra were recorded using KBr discs on FT-IR 1650 Perkin Elmer Spectrophotometer. ^1^H-NMR spectra were recorded in DMF-d_6_ at room temperature using TMS as internal standard on a Bruker 250 MHz spectrophotometer. Magnetic susceptibilities of the complexes were measured by the Gouy method at room temperature using a model MKI Johnson Matthey Alpha Products magnetic susceptibility balance. The effective magnetic moments were calculated using the relation (μeff = 2.828 (χ_m_ T)_½_ B.M. where χ_m_ is the molar susceptibility corrected using pascal's constants for diamagnetism of all atoms in the compounds. Mass spectra were recorded at 70 ev and 300 °C on an MS 5988 Hewlett-packard mass spectrometer. Conductivities were measured in DMF solutions of the complexes (10^−3^ M) using a model LBR, WTWD-812 Wilhelm Conductivity meter fitted with a model LTA 100 cell. Analyses of the metals followed decomposition of their complexes with concentrated nitric acid. The resultant solution was diluted with distilled water, filtered to remove the precipitated ligand. The solution was then neutralized with aqueous ammonia solution and the metal ions titrated with EDTA. Analysis of the uranyl complex was carried out at the Central Laboratory for Environmental Quality Monitoring, CLQM, Kalubia, Cairo, Egypt. The complex was first dried and ground followed by digestion by nitric-HF digestion mixture using Milestone Microwave Digester Model MLS 1200 Mega. The digestible uranium metal was analyzed using Perkin Elmer ICP OES, Model Optima-3000 coupled with an Ultra Sonic Nebulizer, USN. Microanalyses of carbon, hydrogen, nitrogen and chlorine were carried out at the Microanalytical Center, Cairo University, Giza, Egypt. Chlorine in the Fe(III) complexes was determined by ion chromatography using a (IC) Dionex 500 instrument for anions at the Central Laboratory for Environmental Quality Monitoring, EI-Kanater, Cairo, Egypt.

### 3.6. Pharmacology

The *in vitro* evaluation of antimicrobial activity was carried out at Saudi Pharmaceutical Industries and Medical Appliance Corporation. The purpose of the screening program is to provide antimicrobial activity and bacteriostatic andfungistatic efficiency of the investigated metal complexes. The prepared compounds were tested against one strain of Gram +ve bacteria (*Staphylococcus aureu*), Gram −ve bacteria (*Escherichia coli*), and fungi (*Candida albicans*) to provide the MICs (Minimum inhibitory concentration) for each complex. Bacteriostatic and fungistatic efficiency is the lowest concentration of solution to inhibit the growth of a test organism.

## 4. Conclusions

The results of this investigation support the suggested structures of the metal complexes. In case of Cu(II),Ni(II) Co(II), and UO_2_ (IV) cations, only mononuclear complexes were obtained in presence and absence of LiOH indicating that the presence of LiOH does not affect the formation of such complexes. On the other hand, two Fe(III) complexes, mono and binuclear, were obtained in the absence and presence of LiOH, respectively, during their preparation as mention in the Experimental section. All Cu(II), Ni(II), Co(II), and Fe(III) metal cation complexes were octahedral, while Cu(II), and Ni(II) cations in mixed ligand complexes gave an extra square planar and tetrahedral geometries, respectively. Binuclear complex of Fe(III) have octahedral structure in which the two Fe (III) cations are bridged by two chlorine atoms. Table 5 shows that all compounds had high ability to stop and kill microorganisms at 100% concentrations.
